# Supplementary material for: Structure of photosystem I-LHCI-LHCII from the green alga Chlamydomonas reinhardtii in State 2
Source: Nat Commun. 2021 Feb 17;12:1100. doi: 10.1038/s41467-021-21362-6 (PMC7889890; doi:10.1038/s41467-021-21362-6)
Supplement: Supplementary file 1 — Supplementary Information [file 41467_2021_21362_MOESM1_ESM.pdf]

Supplementary Information for

Structure of photosystem I-LHCI-LHCII from the green alga *Chlamydomonas reinhardtii* in State 2

Zihui Huang, Liangliang Shen, Wenda Wang, Zhiyuan Mao, Xiaohan Yi, Tingyun Kuang, Jian-Ren Shen, Xing Zhang, Guangye Han

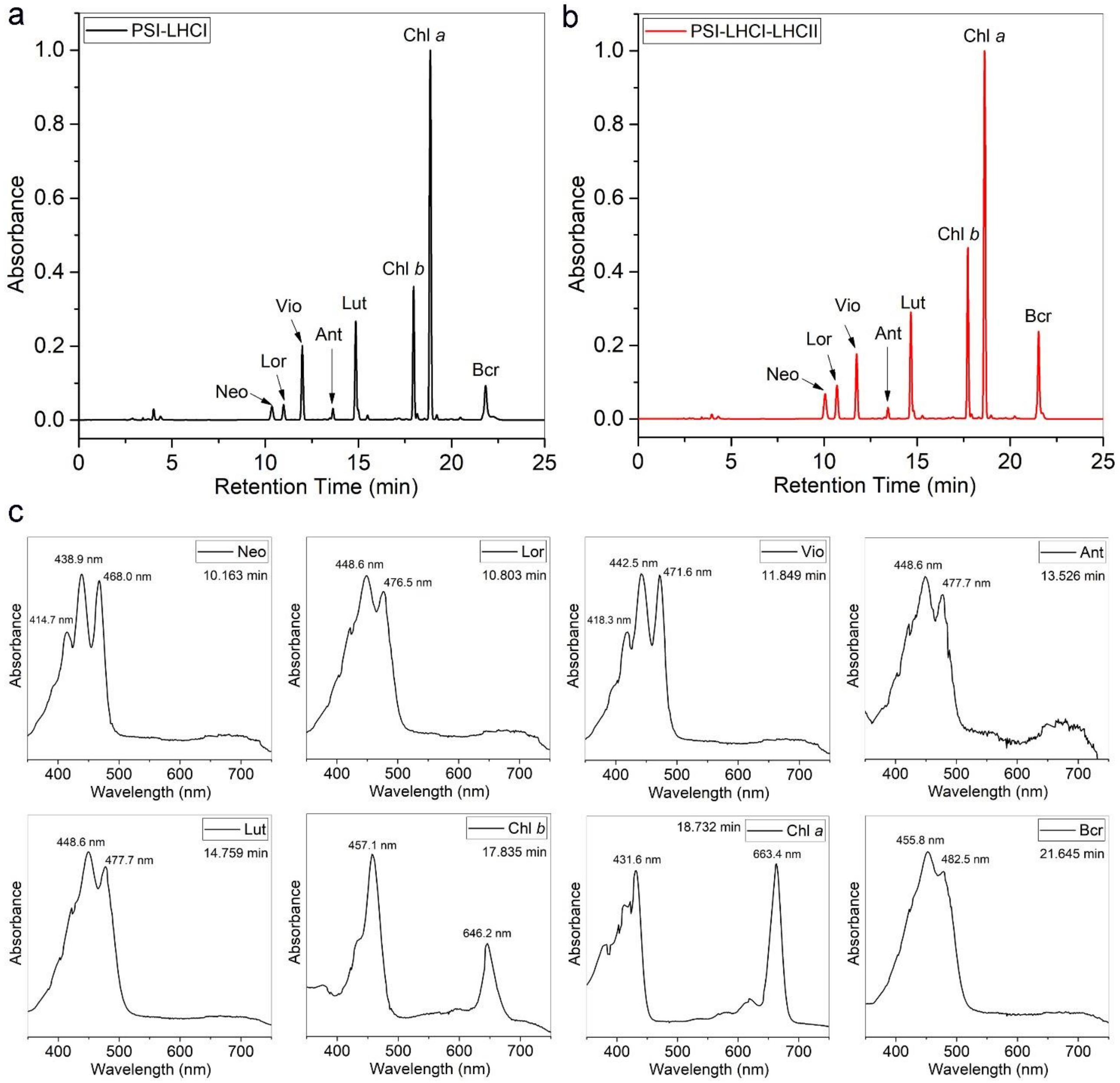

Supplementary Fig. 1. HPLC analysis of pigment content in the PSI-LHCI and PSI-LHCI-LHCII supercomplexes of *C. reinhardtii* obtained from state 2 cells. a, **b**, Pigment composition of PSI-LHCI (**a**) and PSI-LHCI-LHCII (**b**) from state 2 cells of *C. reinhardtii*. Based on the characteristic absorption spectrum and elute time of each fraction, eight major pigment peaks were identified which are neoxanthin (Neo), loroxanthin (Lor), violaxanthin (Vio), antheraxanthin (Ant), lutein (Lut), chlorophyll *b* (Chl *b*), chlorophyll *a* (Chl *a*) and β-carotene (Bcr), respectively. **c**, Room-temperature absorption spectra of different pigments from PSI-LHCI-LHCII of *C. reinhardtii*. The pigment analysis shown in panels **a** and **b** were conducted more than three times, and all showed the same results as shown here.

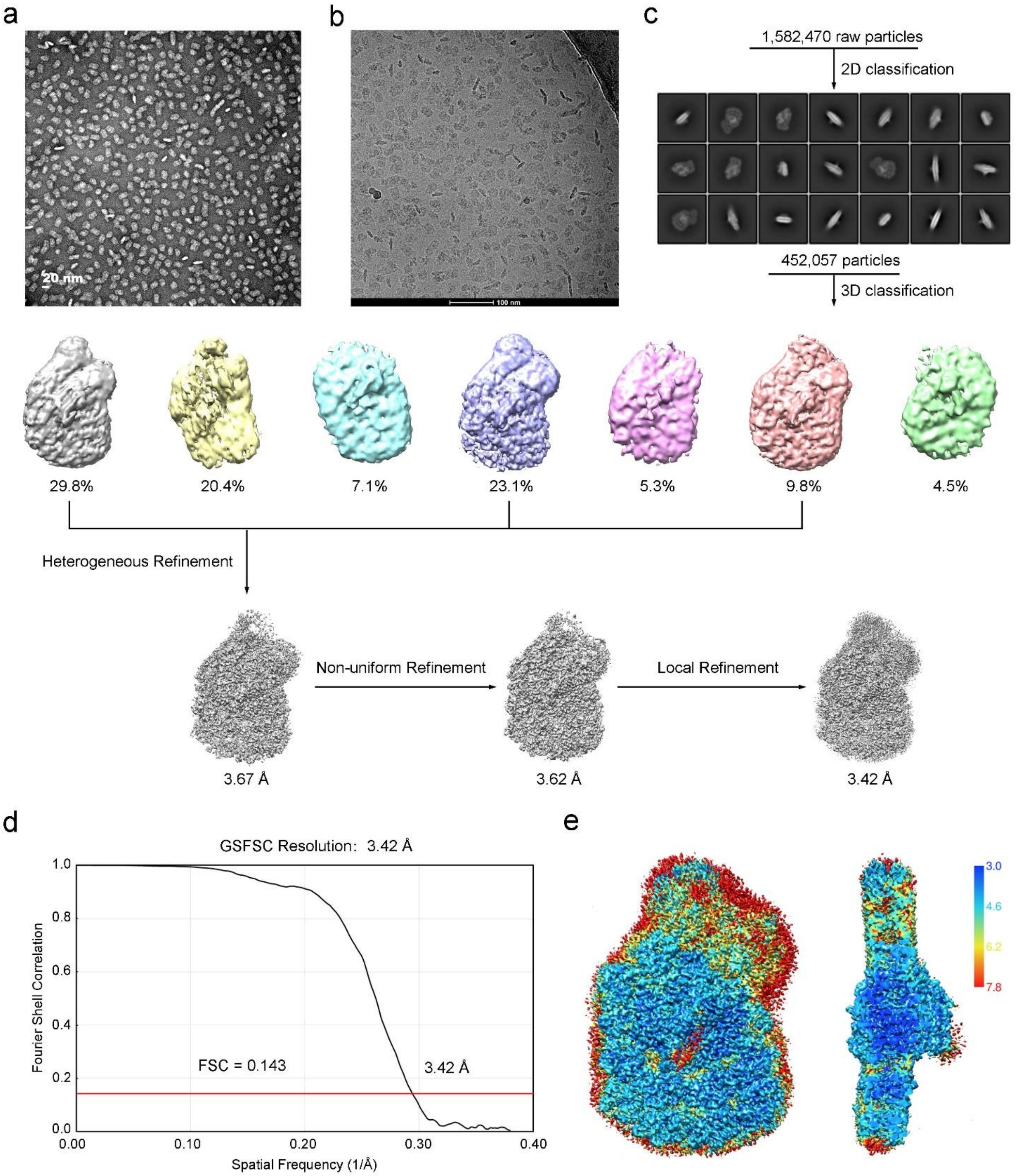

Supplementary Fig. 2. Data collection and image processing. **a**, **b**, Representative of negative staining micrograph and cryo-EM micrograph of the PSI-LHCI-LHCII supercomplex from *C. reinhardtii*. **c**, Flow chart of the cryo-EM data processing of the PSI-LHCI-LHCII supercomplex. **d**, The gold standard FSC curves of the final 3D reconstruction of the PSI-LHCI-LHCII supercomplex. **e**, Local resolution distributions of the PSI-LHCI-LHCII supercomplex generated with RELION. The micrographs in panels **a** and **b** are representatives of more than 100 and 5,087 images, respectively.

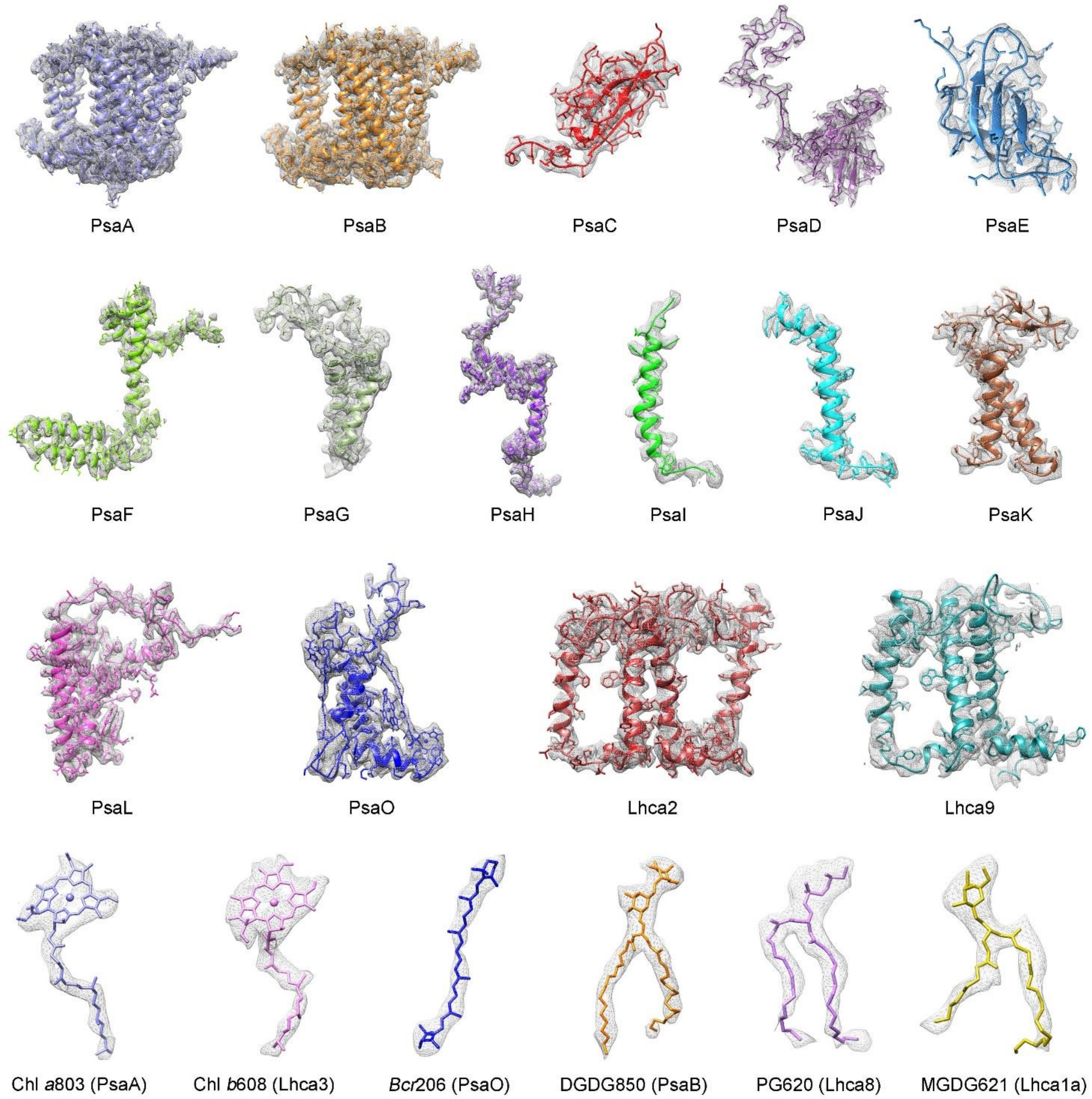

Supplementary Fig. 3. Cryo-EM density map of protein subunits and typical cofactors in the PSI-LHCI-LHCII supercomplex of *C. reinhardtii.* The PSI core and Lhca2/Lhca9 antenna are shown as mixed cartoon/stick and colored as that in Fig.1a. The densities of the pigments in PsaH, PsaL and PsaO were shown together with protein subunits. Pigments (Chl *a*, Chl *b* and Bcr) and lipids (digalactosyldiacylglycerol (DGDG), phosphatidylglycerol (PG) and monogalactosyldiacylglycerol (MGDG)) are represented by sticks. The cryo-EM density maps of each subunit and cofactors are depicted in gray meshes.

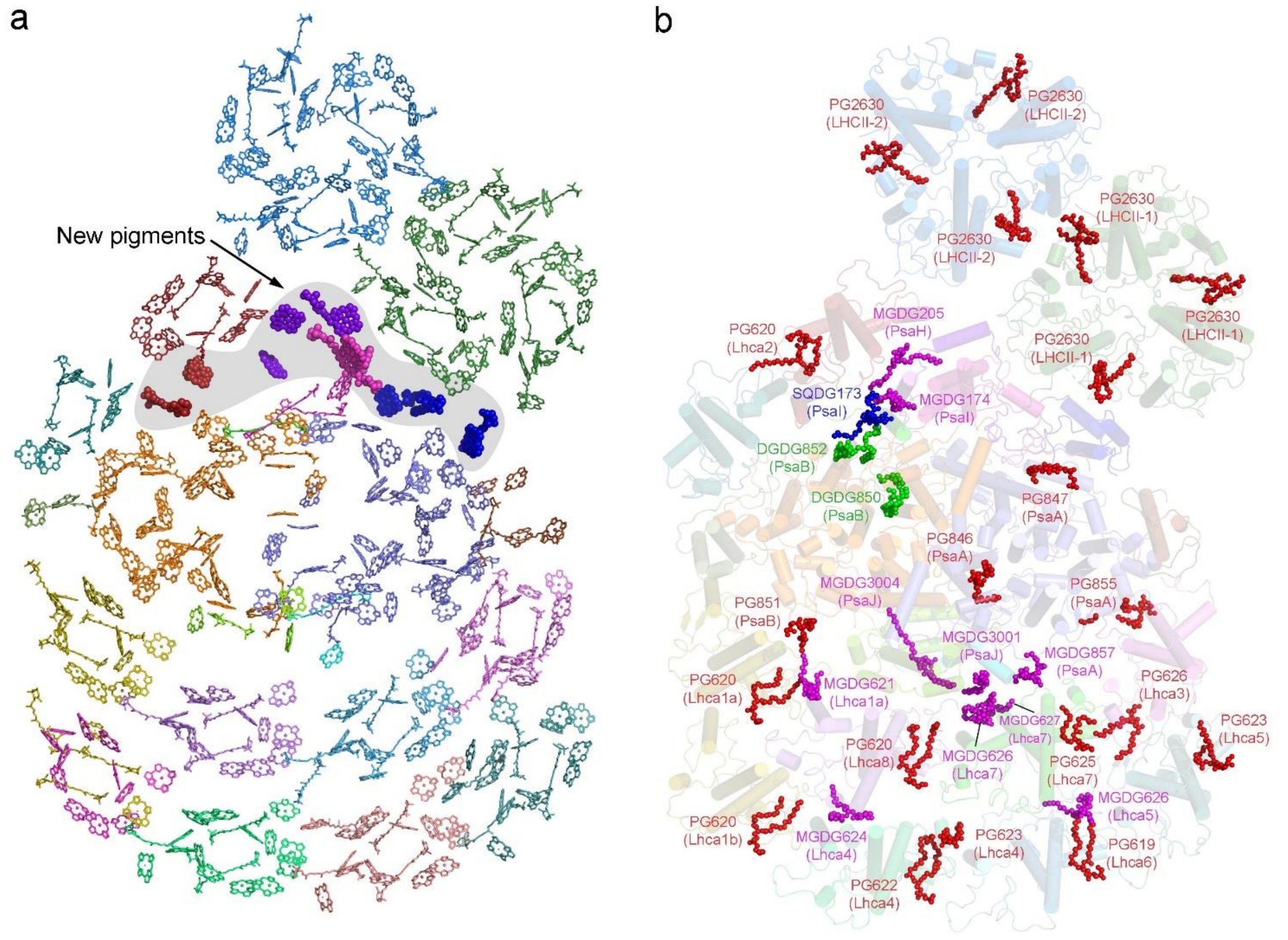

Supplementary Fig. 4. Pigment and lipid distribution in the PSI-LHCI-LHCII supercomplex of *C. reinhardtii*, viewed from the stromal side. a, Pigment arrangement in the PSI-LHCI-LHCII supercomplex. Chlorophylls and carotenoids are shown in stick, and the color corresponds to the color of protein subunits shown in Fig. 1a. The phytol chains of all chlorophylls were deleted for clarity. The newly discovered pigments are shown in spheres. **b**, Distribution of lipids in the green algal PSI-LHCI-LHCII. Each subunit is shown in cartoon with the same color as in Fig. 1a. Four types of lipid molecules [PG (red), DGDG (green), MGDG (magenta), sulfoquinovosyl diacylglycerol (SQDG) (blue)] are depicted in sphere modes.

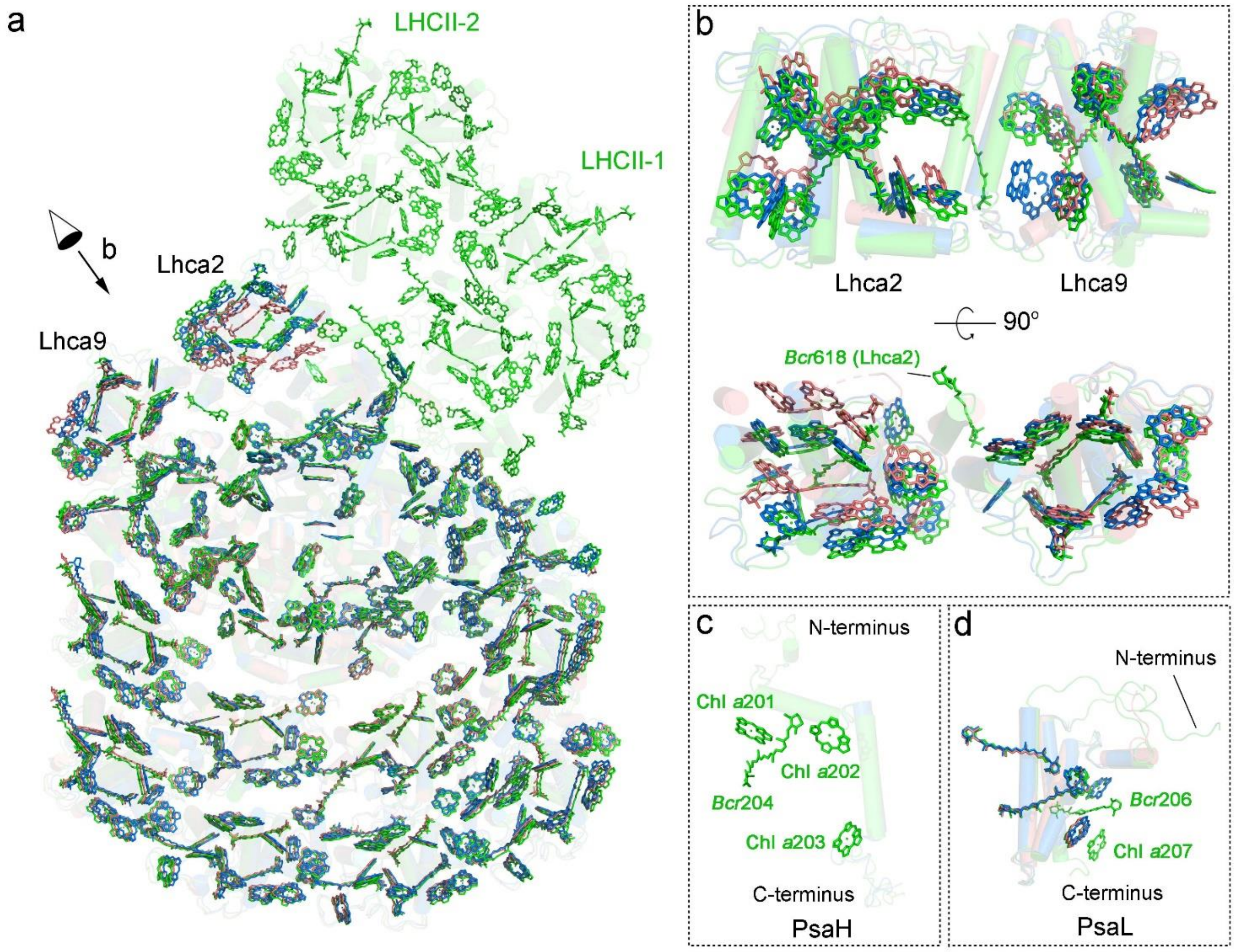

Supplementary Fig. 5. Comparison of all pigments in the PSI-LHCI moieties of the Cr-PSI-LHCI-LHCII with those in the Cr-PSI-LHCI structures (PDB ID: 6JO5[http://doi.org/10.2210/pdb6JO5/pdb]/6IJO[http://doi.org/10.2210/pdb6IJO/pdb]), viewed from the stromal side. **a**, Superposition of all pigment molecules in the PSI-LHCI moieties of three structures, viewed from the stromal side. Differences in pigment distribution in the Lhca2/Lhca9 and interfacial regions between the PSI core and LHCII were found. **b**, Enlarged views of superposition of all pigments in the heterodimer Lhca2/Lhca9 shown in **a**. **c**, Superposition of pigment molecules bound to PsaH from Cr-PSI-LHCI-LHCII and Cr-PSI-LHCI (6IJO). Three Chl *a* (Chl *a*201/Chl *a*202/Chl *a*203) molecules and one molecule of β-carotene (*Bcr*204) were discovered in PsaH from Cr-PSI-LHCI-LHCII. **d**, Superposition of pigment molecules in the PsaL of three structures. One Chl *a* (Chl *a*207) and one β-carotene (*Bcr*206) were newly discovered. These newly discovered pigments are beneficial to the energy transfer from LHCII to PSI core. Color and PDB codes: PSI-LHCI-LHCII, green; 6JO5[http://doi.org/10.2210/pdb6JO5/pdb], deep salmon; 6IJO[http://doi.org/10.2210/pdb6IJO/pdb], marine.

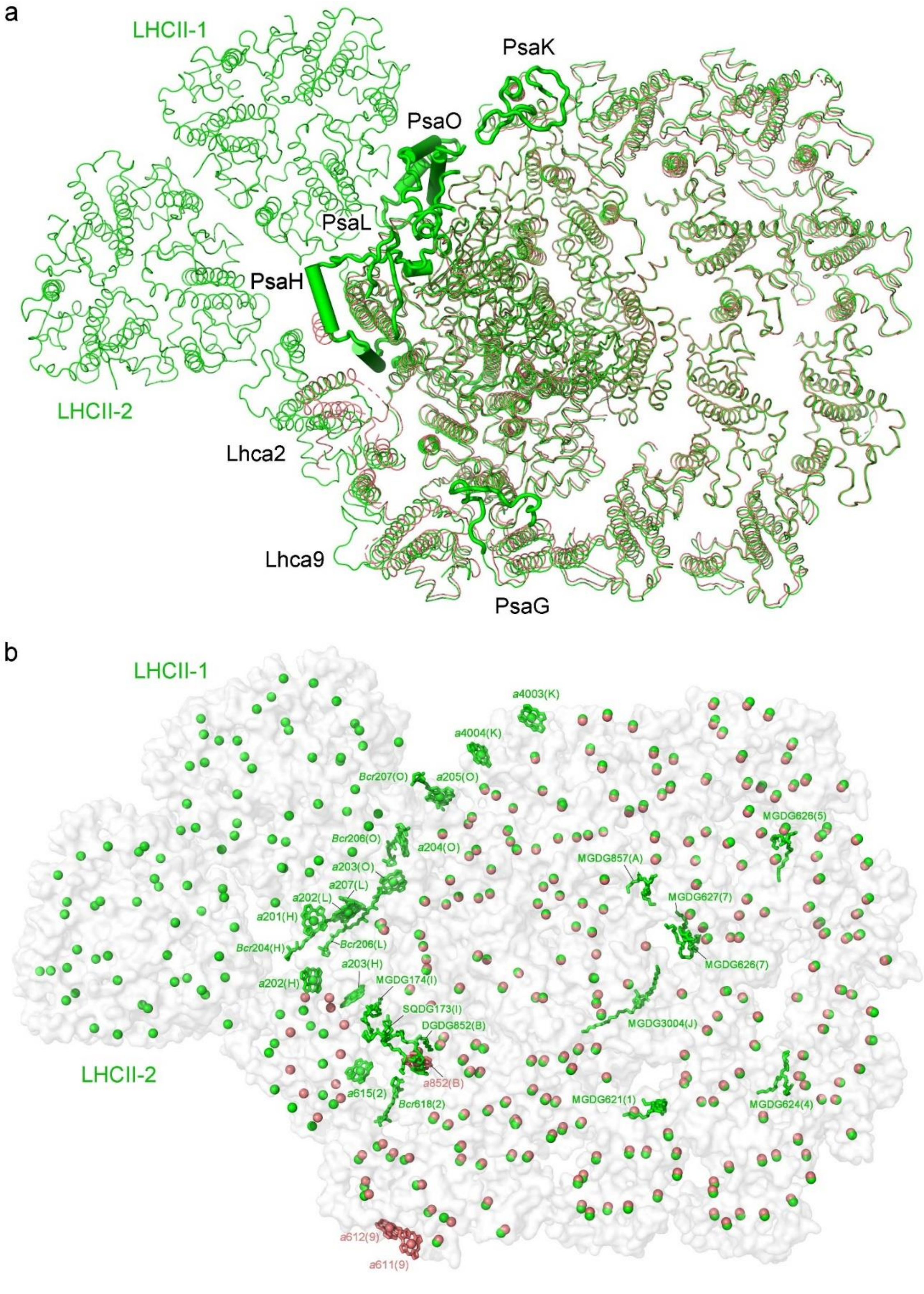
a
LHCII-1
PsaK
PsaO
PsaL
PsaH
LHCII-2
Lhca2
Lhca9
PsaG
b
LHCII-1
a4003(K)
a4004(K)
Bcr207(O)
a205(O)
Bcr206(O)
a204(O)
a203(O)
a207(L)
a202(L)
a201(H)
Bcr204(H)
Bcr206(L)
a203(H)
a202(H)
MGDG174(I)
SQDG173(I)
DGDG852(B)
a852(B)
a615(2)
Bcr618(2)
MGDG626(5)
MGDG857(A)
MGDG627(7)
MGDG626(7)
MGDG3004(J)
MGDG621(1)
MGDG624(4)
LHCII-2
a612(9)
a611(9)

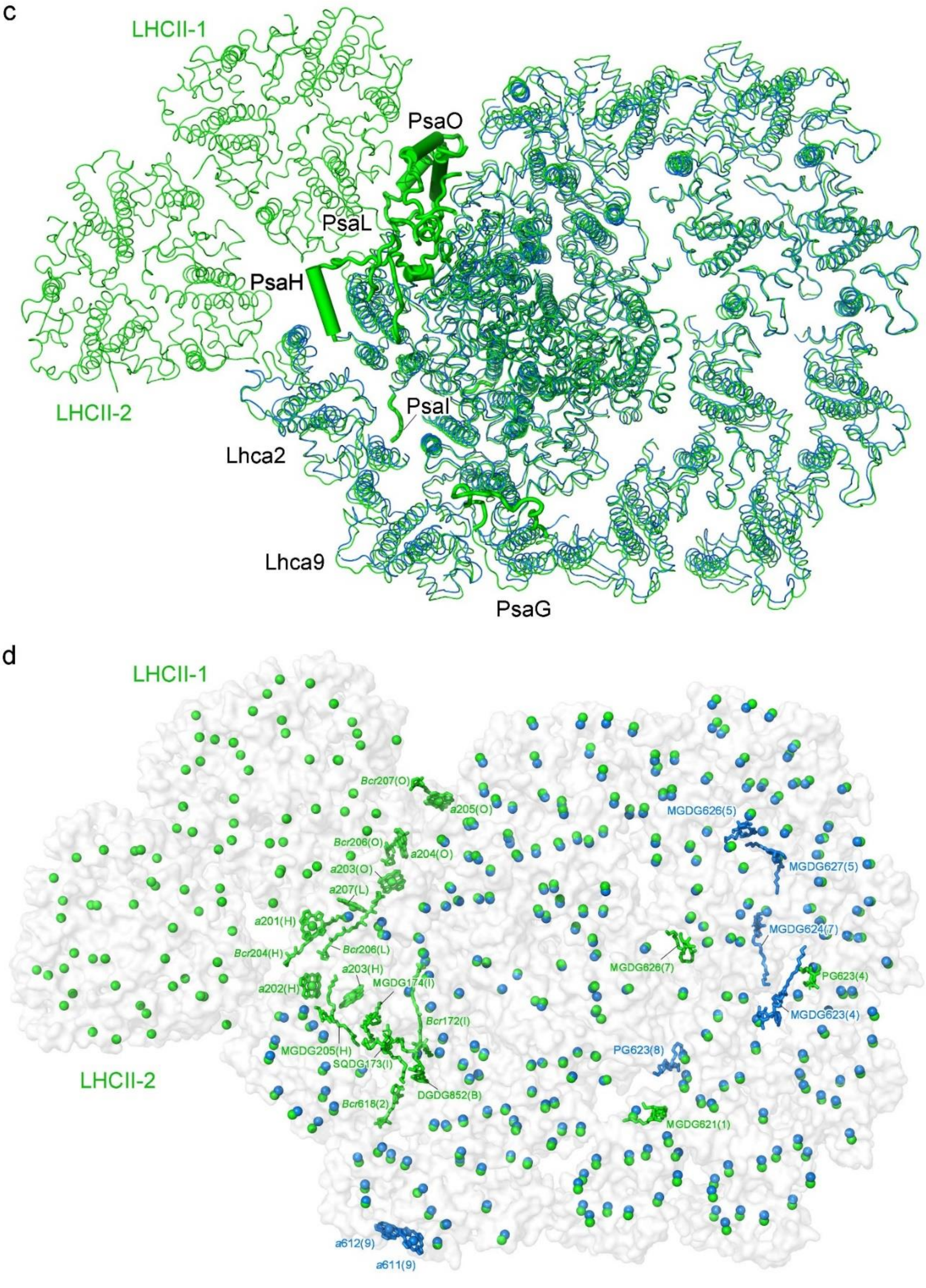

Supplementary Fig. 6. Structural comparison of the PSI-LHCI moieties of the Cr-PSI-LHCI-LHCII with the previously reported Cr-PSI-LHCI structures (PDB ID: 6JO5[http://doi.org/10.2210/pdb6JO5/pdb]/6IJO[http://doi.org/10.2210/pdb6IJO/pdb]), viewed from the stromal side. a, Structural comparison of the PSI-LHCI moieties of Cr-PSI-LHCI-LHCII (green) with the Cr-PSI-LHCI structure (PDB ID: 6JO5[http://doi.org/10.2210/pdb6JO5/pdb]). The newly found PsaH and PsaO subunits in Cr-PSI-LHCI-LHCII are highlighted in cylinder cartoon models and the extra stromal loop regions of PsaL, PsaG and PsaK in the Cr-PSI-LHCI-LHCII

supercomplex are highlighted in bold. The rest of the protein subunits are shown as ribbon models. **b**, Comparison of the pigments and lipids in the PSI-LHCI moieties of Cr-PSI-LHCI-LHCII with those in Cr-PSI-LHCI (PDB ID: 6JO5[http://doi.org/10.2210/pdb6JO5/pdb]). Chlorophylls presented in both Cr-PSI-LHCI-LHCII and Cr-PSI-LHCI are shown as spheres at their Mg positions whereas the newly discovered pigments mainly located in the LHCI or the interfacial region between LHCII and the PSI core in the Cr-PSI-LHCI-LHCII supercomplex are shown in stick models. **c**, Structural comparison of the PSI-LHCI moieties of Cr-PSI-LHCI-LHCII (green) with the Cr-PSI-LHCI structure (PDB ID: 6IJO[http://doi.org/10.2210/pdb6IJO/pdb]). Extra secondary structure regions were observed in the stromal side of PsaH, PsaL and PsaG of the Cr-PSI-LHCI-LHCII complex. A lumenal tail of PsaI was found in the Cr-PSI-LHCI-LHCII. The newly found PsaO are shown in cylinder cartoon. **d**, Comparison of the pigments and lipids in the PSI-LHCI moieties of Cr-PSI-LHCI-LHCII with those in Cr-PSI-LHCI (PDB ID: 6IJO[http://doi.org/10.2210/pdb6IJO/pdb]). Chlorophylls presented in both the Cr-PSI-LHCI-LHCII and Cr-PSI-LHCI are shown as spheres at their Mg positions whereas the newly discovered pigments mainly located in the interfacial region between LHCII/LHCI and the PSI core in the Cr-PSI-LHCI-LHCII supercomplex are shown in stick models. The varied lipids are shown in thick stick models. Color codes: PSI-LHCI-LHCII, green; 6JO5[http://doi.org/10.2210/pdb6JO5/pdb], deep salmon; 6IJO[http://doi.org/10.2210/pdb6IJO/pdb], marine.

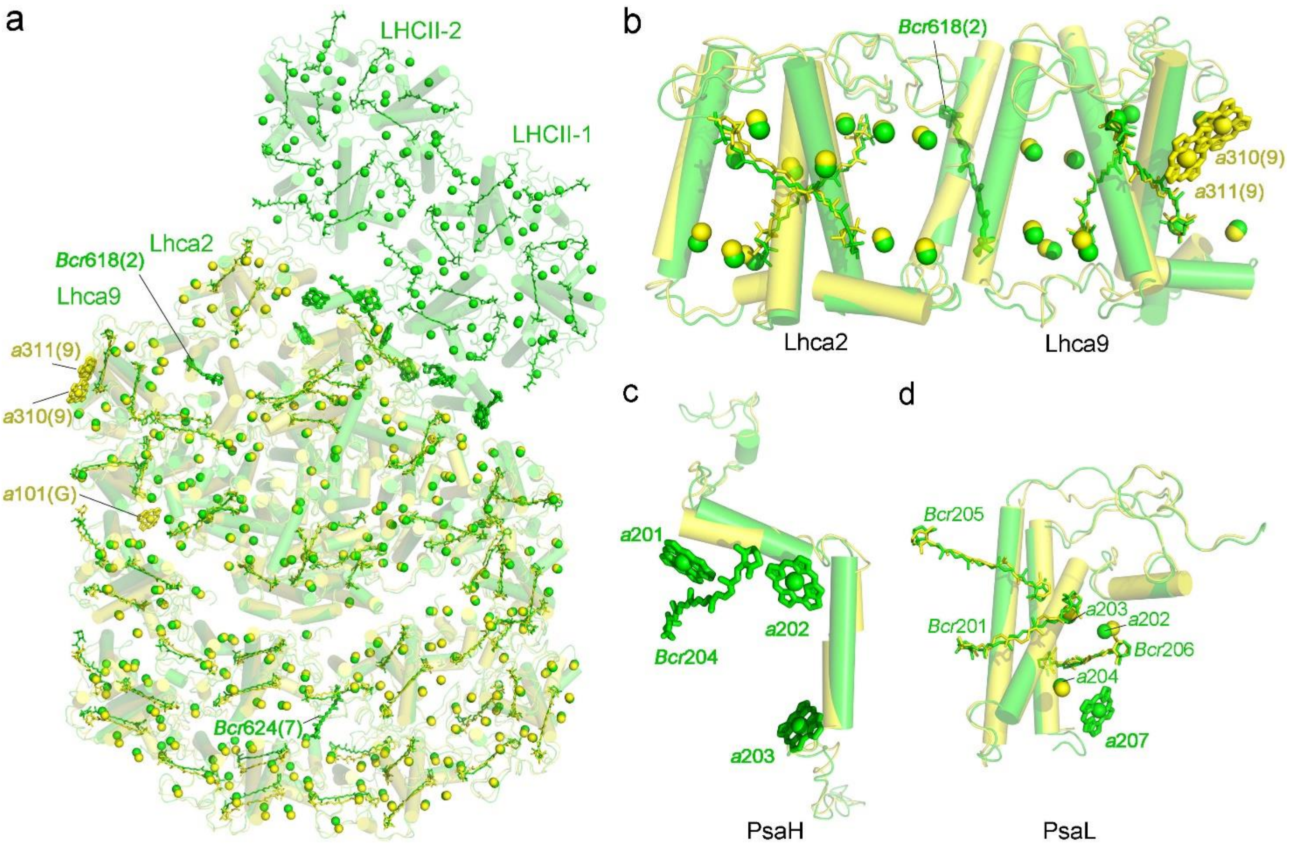

Supplementary Fig. 7. Structural comparison of PSI-LHCI-LHCII from *C. reinhardtii* with PSI-LHCI from the green alga (*B. corticulans*). a, Structural superposition of Cr-PSI-LHCI-LHCII (green) with the PSI-LHCI supercomplex of *B. corticulans* (PDB code 6IGZ[http://doi.org/10.2210/pdb6IGZ/pdb], yellow) viewed from the stromal side. **b**-**d**, Enlarged views of the Lhca2/Lhca9 subunits (**b**), PsaH subunits (**c**) and PsaL subunits (**d**) in **a**. Two new chlorophylls were located in Lhca9 of *B. corticulans* compared with that of *C. reinhardtii*. Three chlorophylls and one β-carotene in the interfacial region between LHCII and PSI core were discovered in PsaH of *C. reinhardtii* compared to that of *B. corticulans*. One chlorophyll (Chl *a*207) was discovered in the PsaL of *C. reinhardtii* compared to that of *B. corticulans*. The protein subunits are shown as cartoon models. Conserved chlorophylls are shown as spheres at their Mg positions whereas the newly discovered chlorophylls, carotenoids and lipids are shown in thick sticks. Color codes: PSI-LHCI-LHCII, green; 6IGZ[http://doi.org/10.2210/pdb6IGZ/pdb], yellow.

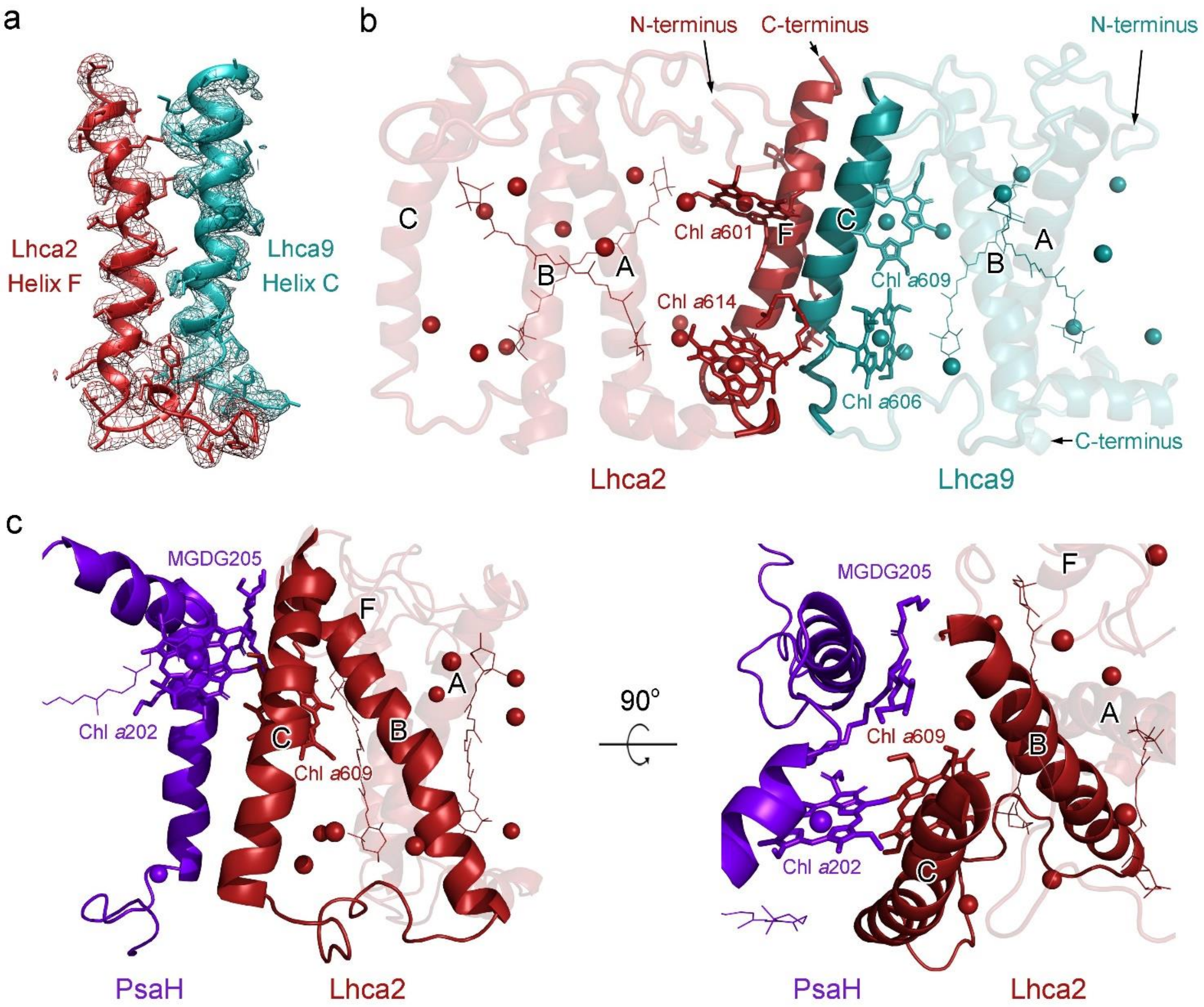

Supplementary Fig. 8. The interactions between the Lhca2 and Lhca9, and between Lhca2 and PsaH in the PSI-LHCI-LHCII supercomplex of *C. reinhardtii*. **a**, The cryo-EM densities of the helix F of Lhca2 and helix C of Lhca9, and the interface between Lhca2 and Lhca9. **b**, The interactions between Lhca2 and Lhca9. The interactions between helix-F of Lhca2 and helix C of Lhca9, as well as the adjacent chlorophylls in these subunits are highlighted whereas the rest of subunits are shown as transparent cartoon models. **c**, The interactions between Lhca2 and PsaH.

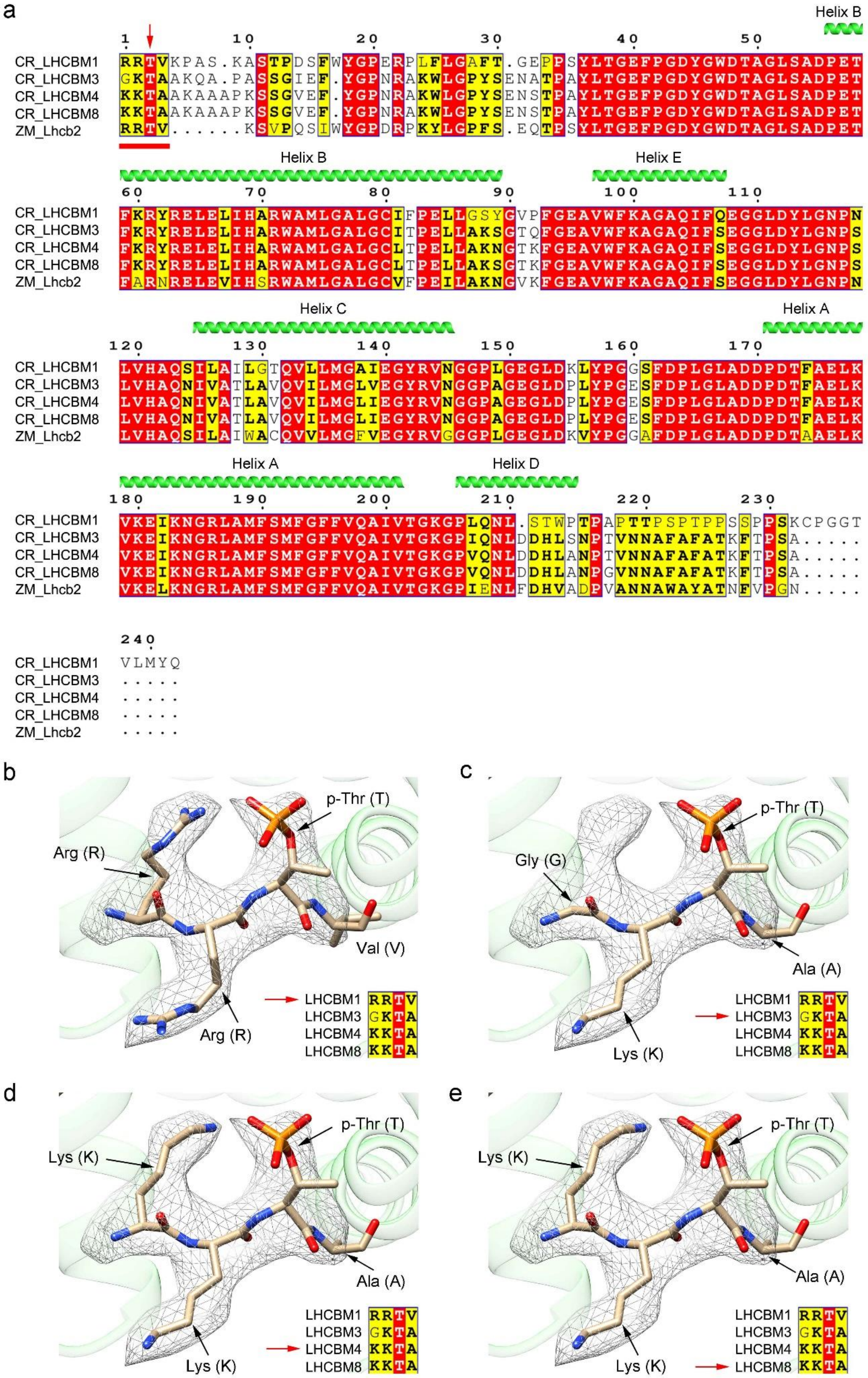
a
Helix B
Helix E
Helix C
Helix A
Helix D
CR_LHCBM1 RRTVKPAS.KASTPDSFWYGPERPLFLGAFT.GEPPSYLTGEFPGDYGWDTAGLSADPET
CR_LHCBM3 GKTAAKQA.PASSGIEF.YGPNRAKWLGPYSENATPAYLTGEFPGDYGWDTAGLSADPET
CR_LHCBM4 KKTAAKAAAPKSSGVEF.YGPNRAKWLGPYSENSTPAYLTGEFPGDYGWDTAGLSADPET
CR_LHCBM8 KKTAAKAAAPKSSGVEF.YGPNRAKWLGPYSENSTPAYLTGEFPGDYGWDTAGLSADPET
ZM_Lhcb2 RRTV......KSVPQSIWYGPDRPKYLGPFS.EQTPSYLTGEFPGDYGWDTAGLSADPET
CR_LHCBM1 FKRYRELELIHARWAMLGALGCIFPELLGSYGVPFGEAVWFKAGAQIFQEGGLDYLGNPN
CR_LHCBM3 FKRYRELELIHARWAMLGALGCITPELLAKSGTQFGEAVWFKAGAQIFSEGGLDYLGNPS
CR_LHCBM4 FKRYRELELIHARWAMLGALGCLTPELLAKNGTKFGEAVWFKAGAQIFSEGGLDYLGNPS
CR_LHCBM8 FKRYRELELIHARWAMLGALGCLTPELLAKSGTKFGEAVWFKAGAQIFSEGGLDYLGNPS
ZM_Lhcb2 FARNRELEVIHSRWAMLGALGCVFPEILAKNGVKFGEAVWFKAGAQIFSEGGLDYLGNPN
CR_LHCBM1 LVHAQSILAILGTQVLLMGAIEGYRVNGGPLGEGLDKLYPGGSFDPLGLADDPDTFAELK
CR_LHCBM3 LVHAQNIVATLAVQVILMGLVEGYRVNGGPAGEGLDPLYPGESFDPLGLADDPDTFAELK
CR_LHCBM4 LVHAQNIVATLAVQVILMGLIEGYRVNGGPAGEGLDPLYPGESFDPLGLADDPDTFAELK
CR_LHCBM8 LVHAQNIVATLAVQVILMGLIEGYRVNGGPAGEGLDPLYPGESFDPLGLADDPDTFAELK
ZM_Lhcb2 LVHAQSILAIWACQVVLMGFVEGYRVGGGPLGEGLDKVYPGGAFDPLGLADDPDTAAELK
CR_LHCBM1 VKEIKNGRLAMFSMFGFFVQAIVTGKGPLQNL.STWPTPAPTTPSPTPPSSPPSKCPGGT
CR_LHCBM3 VKEIKNGRLAMFSMFGFFVQAIVTGKGPIQNLDDHLSNPTVNNAFAFATKFTPSA.....
CR_LHCBM4 VKEIKNGRLAMFSMFGFFVQAIVTGKGPVQNLDDHLANPTVNNAFAFATKFTPSA.....
CR_LHCBM8 VKEIKNGRLAMFSMFGFFVQAIVTGKGPVQNLDDHLANPGVNNAFAFATKFTPSA.....
ZM_Lhcb2 VKELKNGRLAMFSMFGFFVQAIVTGKGPIENLFDHVADPVANNAWAYATNFVPGN.....
CR_LHCBM1 VLMYQ
CR_LHCBM3 .....
CR_LHCBM4 .....
CR_LHCBM8 .....
ZM_Lhcb2 .....
b
p-Thr (T)
Arg (R)
Val (V)
Arg (R)
c
p-Thr (T)
Gly (G)
Ala (A)
Lys (K)
d
p-Thr (T)
Lys (K)
Ala (A)
Lys (K)
e
p-Thr (T)
Lys (K)
Ala (A)
Lys (K)
LHCBM1 RRTV
LHCBM3 GKTA
LHCBM4 KKTA
LHCBM8 KKTA

Supplementary Fig. 9. Sequence alignment of Lhcb between LHCBM1/LHCBM3/4/8 from *C. reinhardtii* and Lhcb2 from maize (PDB code: 5ZJI[http://doi.org/10.2210/pdb5ZJI/pdb]) to identify the phosphorylated LHCBM subunit involved in the interactions with the PSI core subunits in Cr-PSI-LHCI-LHCII. a, Sequence alignment of LHCBM1/LHCBM3/4/8 from *C. reinhardtii* with the Lhcb2 from maize. The red arrow shows the phosphorylated residue Thr from maize Lhcb2 and that identified in both LHCBM1 and LHCBM3/4/8 based on the experiments for phosphorylation identification. **b**, **c**, **d, e** Assignment of the LHCBM subunit involved in the interactions with the PSI core subunits by comparison of the structural features among amino acids of Arg (**b**), Gly (**c**) and Lys (**d, e**), the initial residue of LHCBM1 and LHCBM3/4/8, respectively. The possibility of LHCBM3 as the LHCBM subunit involved in the interactions with the PSI core subunits in Cr-PSI-LHCI-LHCII is excluded on the basis of (**b**) and (**c**). The sequences used are: LHCBM1[https://www.ncbi.nlm.nih.gov/gene/?term=lhcbm1], LHCBM3[https://www.ncbi.nlm.nih.gov/gene/?term=lhcbm3], LHCBM4[https://www.ncbi.nlm.nih.gov/gene/5720970], LHCBM8[https://www.ncbi.nlm.nih.gov/gene/?term=LHCBM8], ZM_LHCB2[https://www.ncbi.nlm.nih.gov/gene/?term=103643653].

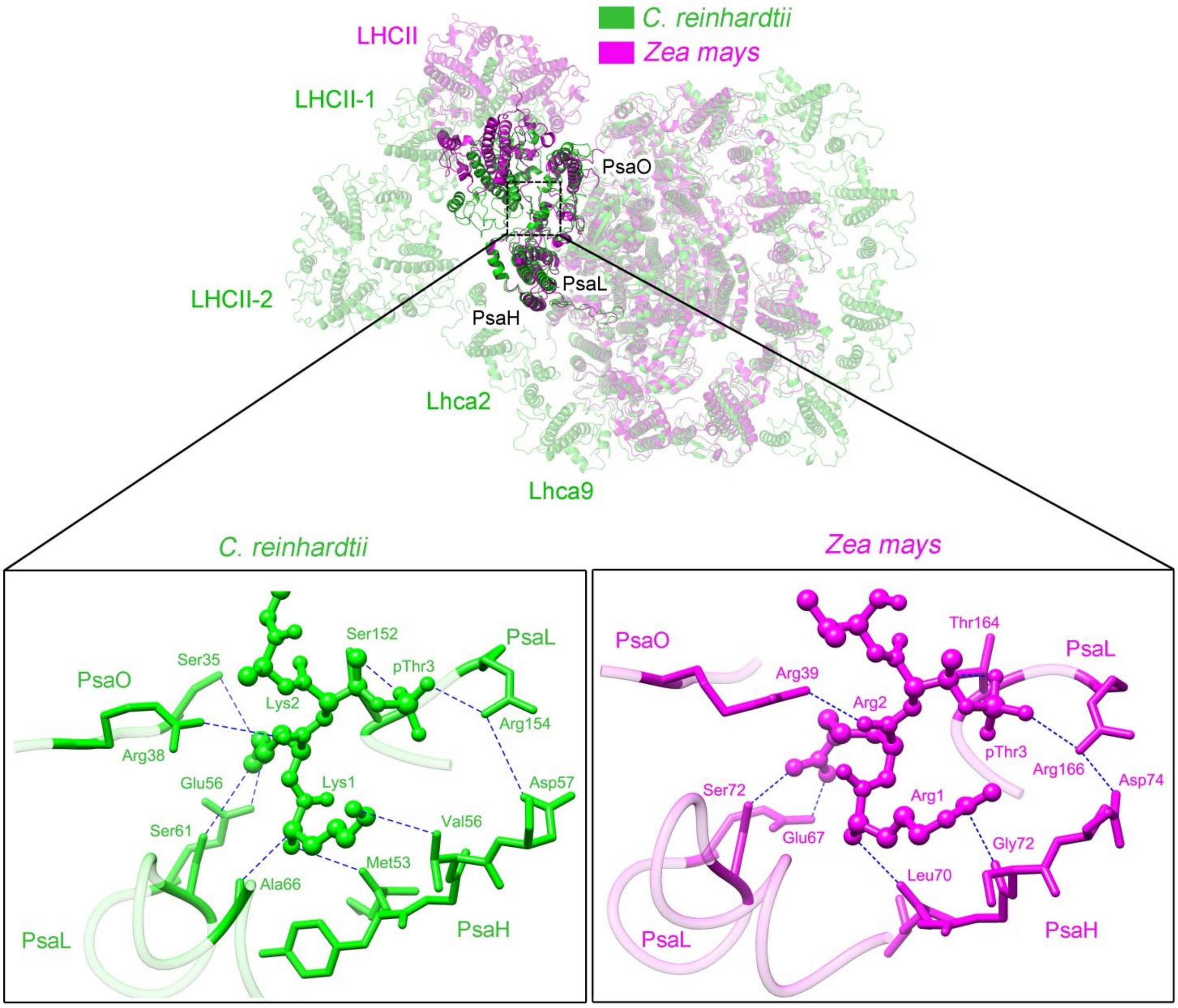

Supplementary Fig. 10. Conformation comparison of the binding site of pThr from LHCII isomer in both Cr-PSI-LHCI-LHCII (green) and maize PSI-LHCI-LHCII (PDB code 5ZJI[http://doi.org/10.2210/pdb5ZJI/pdb], magenta). The LHCII residues involved in the interactions with PSI core subunits are shown in ball and stick, and the residues of PSI core subunits involved in the interactions with the phosphorylated LHCII are shown as sticks. The dashed lines indicate hydrogen bonds or van der Waals interactions between adjacent groups.

Supplementary Table 1. Statistics of structural analysis of the PSI-LHCI-LHCII supercomplex refined against the 3.42 Å resolution cryo-EM map.

	PSI-LHCI-LHCII (EMDB-30536) (PDB 7D0J)
**Data collection and processing**	
Magnification	22,500 ×
Voltage (kV)	300
Electron exposure (e–/Å^2)	50
Defocus range (μm)	-1.8 to -2.3
Pixel size (Å)	1.307
Symmetry imposed	C1
Initial particle images (no.)	452,057
Final particle images (no.)	283,763
Map resolution (Å)	3.42
FSC threshold	0.143
**Refinement**	
Initial model used (PDB code)	6JO5
Map sharpening B factor (Å^2)	81.74
Model composition	
Non-hydrogen atoms	69,243
Protein residues	5,924
Ligands	453
B factors (Å^2)	
Protein	78.16
Ligand	93.33
R.m.s. deviations	
Bond lengths (Å)	0.010
Bond angles (°)	1.750
Validation	
MolProbity score	2.24
Clashscore	15.55
Poor rotamers (%)	0.02
Ramachandran plot	
Favored (%)	90.33
Allowed (%)	9.42
Disallowed (%)	0.26

Supplementary Table 2. Numbers of cofactors in the structural model of the PSI-LHCI-LHCII supercomplex from *C. reinhardtii*.

Subunit	Traced residues	Chlorophylls	Carotenoids	Lipids	Others
PsaA	740 (12-751)	45 Chl *a*	6 Bcr	3 PG<br>1 MGDG	1 PQN<br>1 SF4
PsaB	734 (2-735)	40 Chl *a*	7 Bcr	1 PG<br>2 DGDG	1 PQN
PsaC	80 (2-81)				2 SF4
PsaD	144 (53-196)				
PsaE	63 (34-96)				
PsaF	165 (63-227)	3 Chl *a*	1 Bcr		
PsaG	91 (32-122)	2 Chl *a*	1 Bcr		
PsaH	100 (31-130)	3 Chl *a*	1 Bcr	1 MGDG	
PsaI	37 (68-104)		1 Bcr	1 SQDG<br>1 MGDG	
PsaJ	41 (1-41)	1 Chl *a*	1 Bcr	2 MGDG	
PsaK	85 (29-113)	4 Chl *a*	2 Bcr		
PsaL	159 (38-196)	4 Chl *a*	3 Bcr		
PsaO	93 (31-123)	3 Chl *a*	2 Bcr		
Lhca1a	194 (35-228)	12 Chl *a*<br>2 Chl *b*	3 Lut	1 PG<br>1 MGDG	
Lhca1b	194 (35-228)	12 Chl *a*<br>2 Chl *b*	3 Lut	1 PG	
Lhca2	201 (28-228)	13 Chl *a*	2 Lut<br>1 Bcr	1 PG	
Lhca3	203 (61-263)	13 Chl *a*<br>1 Chl *b*	2 Lut<br>3 Bcr	1 PG	
Lhca4	205 (58-262)	11 Chl *a*<br>4 Chl *b*	2 Lut<br>1 Bcr	2 PG<br>1 MGDG	
Lhca5	225 (32-256)	14 Chl *a*<br>3 Chl *b*	2 Lut<br>2 Bcr	1 PG<br>1 MGDG	
Lhca6	230 (28-257)	13 Chl *a*<br>4 Chl *b*	2 Lut<br>2 Bcr	1 PG	
Lhca7	213 (29-241)	14 Chl *a*<br>1 Chl *b*	2 Lut<br>2 Bcr	1 PG<br>2 MGDG	
Lhca8	217 (27-243)	13 Chl *a*<br>1 Chl *b*	2 Lut<br>1 Bcr	1 PG	
Lhca9	182 (29-210)	8 Chl *a*<br>2 Chl *b*	2 Lut		
LHCII-1<br>(Monomer-1)	233 (21-253)	8 Chl *a*<br>6 Chl *b*	2 Lut<br>1 Vio<br>1 Neo	1 PG	
LHCII-1<br>(Monomer-2 and<br>Monomer-3)	438 (30-248)	16 Chl *a*<br>12 Chl *b*	4 Lut<br>2 Vio<br>2 Neo	2 PG	
LHCII-2<br>(Trimer)	657 (30-248)	24 Chl *a*<br>18 Chl *b*	6 Lut<br>3 Vio<br>3 Neo	3 PG	
Total	5924	332	83	33	5

Abbreviations used: Bcr, β-carotene; Lut, lutein; Neo, neoxanthin; Vio, violaxanthin; MGDG, monogalactosyldiacyl glycerol; SQDG, sulfoquinovosyldiacyl glycerol; DGDG, digalactosyldiacyl glycerol; PG, phosphatidyl glycerol; PQN, phylloquinone; SF4, sulphur-iron cluster.
