# Supplementary material for: Structure of photosystem I-LHCI-LHCII from the green alga Chlamydomonas reinhardtii in State 2
Source: Nat Commun. 2021 Feb 17;12:1100. doi: 10.1038/s41467-021-21362-6 (PMC7889890; doi:10.1038/s41467-021-21362-6)
Supplement: Supplementary file 3 — Reporting Summary [file 41467_2021_21362_MOESM3_ESM.pdf]

nature research

Corresponding author(s): Jian-Ren Shen

Last updated by author(s): Jan 18, 2021

Reporting Summary

Nature Research wishes to improve the reproducibility of the work that we publish. This form provides structure for consistency and transparency in reporting. For further information on Nature Research policies, see our Editorial Policies and the Editorial Policy Checklist.

Statistics

For all statistical analyses, confirm that the following items are present in the figure legend, table legend, main text, or Methods section.

n/a	Confirmed	
☐	☒	The exact sample size (*n*) for each experimental group/condition, given as a discrete number and unit of measurement
☐	☒	A statement on whether measurements were taken from distinct samples or whether the same sample was measured repeatedly
☒	☐	The statistical test(s) used AND whether they are one- or two-sided<br>*Only common tests should be described solely by name; describe more complex techniques in the Methods section.*
☒	☐	A description of all covariates tested
☒	☐	A description of any assumptions or corrections, such as tests of normality and adjustment for multiple comparisons
☒	☐	A full description of the statistical parameters including central tendency (e.g. means) or other basic estimates (e.g. regression coefficient) AND variation (e.g. standard deviation) or associated estimates of uncertainty (e.g. confidence intervals)
☒	☐	For null hypothesis testing, the test statistic (e.g. *F*, *t*, *r*) with confidence intervals, effect sizes, degrees of freedom and *P* value noted<br>*Give P values as exact values whenever suitable.*
☒	☐	For Bayesian analysis, information on the choice of priors and Markov chain Monte Carlo settings
☒	☐	For hierarchical and complex designs, identification of the appropriate level for tests and full reporting of outcomes
☒	☐	Estimates of effect sizes (e.g. Cohen's *d*, Pearson's *r*), indicating how they were calculated

Our web collection on statistics for biologists contains articles on many of the points above.

Software and code

Policy information about availability of computer code

Data collection	serialEM 3.8
Data analysis	Proteome Discovery searching algorithm 1.4, SerialEM 3.8, cryoSparc 2.15, MotionCor2 1.1.0, EMAN 2.2.2, Gctf 1.06, RELION 3.0, Coot 0.8.9, Pymol 2.2.0, Chimera 1.14, Phenix 1.18, PyMOL 2.1.1

For manuscripts utilizing custom algorithms or software that are central to the research but not yet described in published literature, software must be made available to editors and reviewers. We strongly encourage code deposition in a community repository (e.g. GitHub). See the Nature Research guidelines for submitting code & software for further information.

Data

Policy information about availability of data

All manuscripts must include a data availability statement. This statement should provide the following information, where applicable:
- Accession codes, unique identifiers, or web links for publicly available datasets
- A list of figures that have associated raw data
- A description of any restrictions on data availability

The cryo-EM density map and atomic models have been deposited in the Electron Microscopy Data Bank and the Protein Data Bank (EMD ID code 30536 and PDB ID code 7D0J). The data that support the findings of this study are available from the corresponding authors upon reasonable request.

Field-specific reporting

Please select the one below that is the best fit for your research. If you are not sure, read the appropriate sections before making your selection.

☒ Life sciences ☐ Behavioural & social sciences ☐ Ecological, evolutionary & environmental sciences

For a reference copy of the document with all sections, see nature.com/documents/nr-reporting-summary-flat.pdf

Life sciences study design

All studies must disclose on these points even when the disclosure is negative.

Sample size	No statistic method was used to predetermine the sample size. Amount of cryo-EM micrographs collected was based on the previous knowledge and the results indicated that the size is sufficient to generate a density map at 3.42 Å resolution.
Data exclusions	No data exclusion criteria was pre-established. 2D and 3D classification were used to obtain multiple classes. Only the particles in the classes that showed clear structural signals and intact structures were chosen, combined and used in the final reconstruction of cryo-EM map and refinement. Details are described in the flowchart of Supplementary Fig. 4. and Methods.
Replication	Multiple rounds of structural refinement have been performed and all resulted in same density maps, although at different resolutions. Most experiments concerning the purification and characterization of PSI-LHCI-LHCII have been repeated independently for more than 3 times, and all successfully reproduced.
Randomization	Randomization is not relevant to our study because we aimed to solve the structure of the specific protein supercomplex.
Blinding	Blinding is not relevant to our study because we aimed to solve the structure of the specific protein supercomplex.

Reporting for specific materials, systems and methods

We require information from authors about some types of materials, experimental systems and methods used in many studies. Here, indicate whether each material, system or method listed is relevant to your study. If you are not sure if a list item applies to your research, read the appropriate section before selecting a response.

Materials & experimental systems

n/a	Involved in the study
☐	☒ Antibodies
☒	☐ Eukaryotic cell lines
☒	☐ Palaeontology and archaeology
☒	☐ Animals and other organisms
☒	☐ Human research participants
☒	☐ Clinical data
☒	☐ Dual use research of concern

Methods

n/a	Involved in the study
☒	☐ ChIP-seq
☒	☐ Flow cytometry
☒	☐ MRI-based neuroimaging

Antibodies

Antibodies used	The Primary antibody p-Thr antibody was purchased from Cell Signaling Technology (Cat#9386) ;The horseradish peroxidase-conjugated secondary antibody (Goat Anti Mouse IgG (H+L)-HRP) was purchased from Gene Protein Link (Cat#P03501S).
Validation	The Primary antibody p-Thr antibody has been validated by immunoblotting for several times.
