# Supplementary figures and images for: Structure of photosystem I-LHCI-LHCII from the green alga Chlamydomonas reinhardtii in State 2
Source: Nat Commun. 2021 Feb 17;12:1100. doi: 10.1038/s41467-021-21362-6 (PMC7889890; doi:10.1038/s41467-021-21362-6)

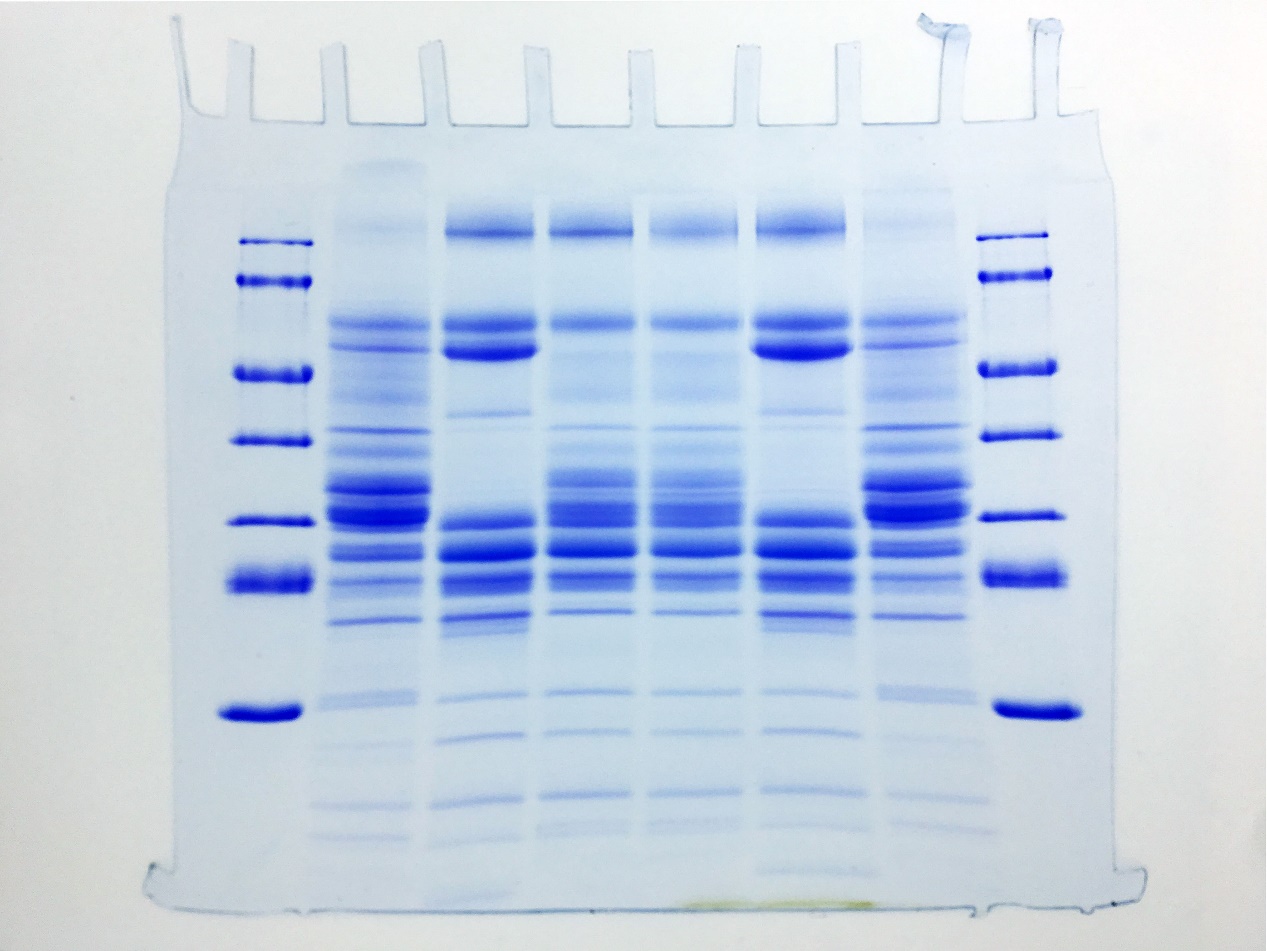


Figure 2a


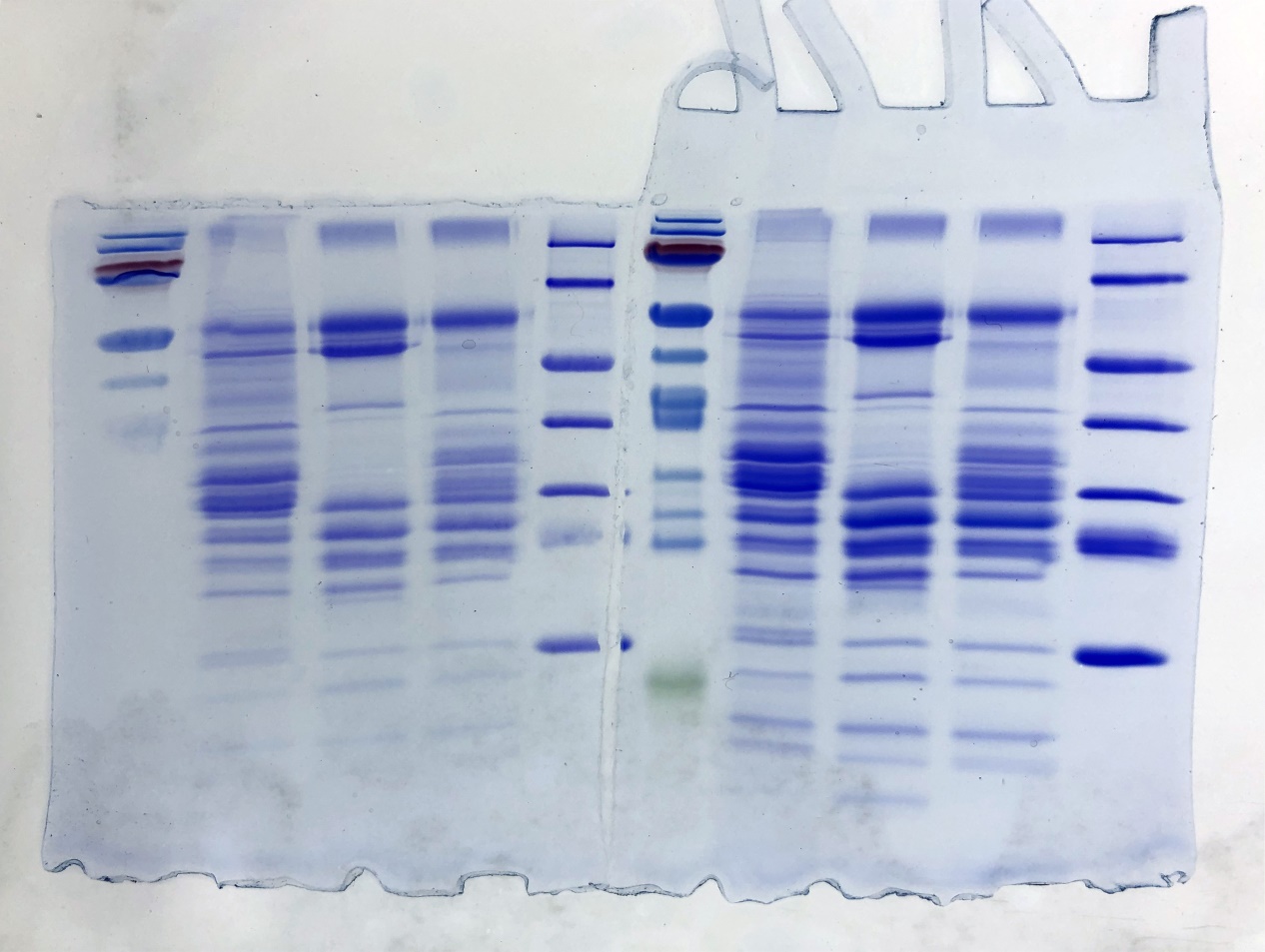


Figure 2b-1


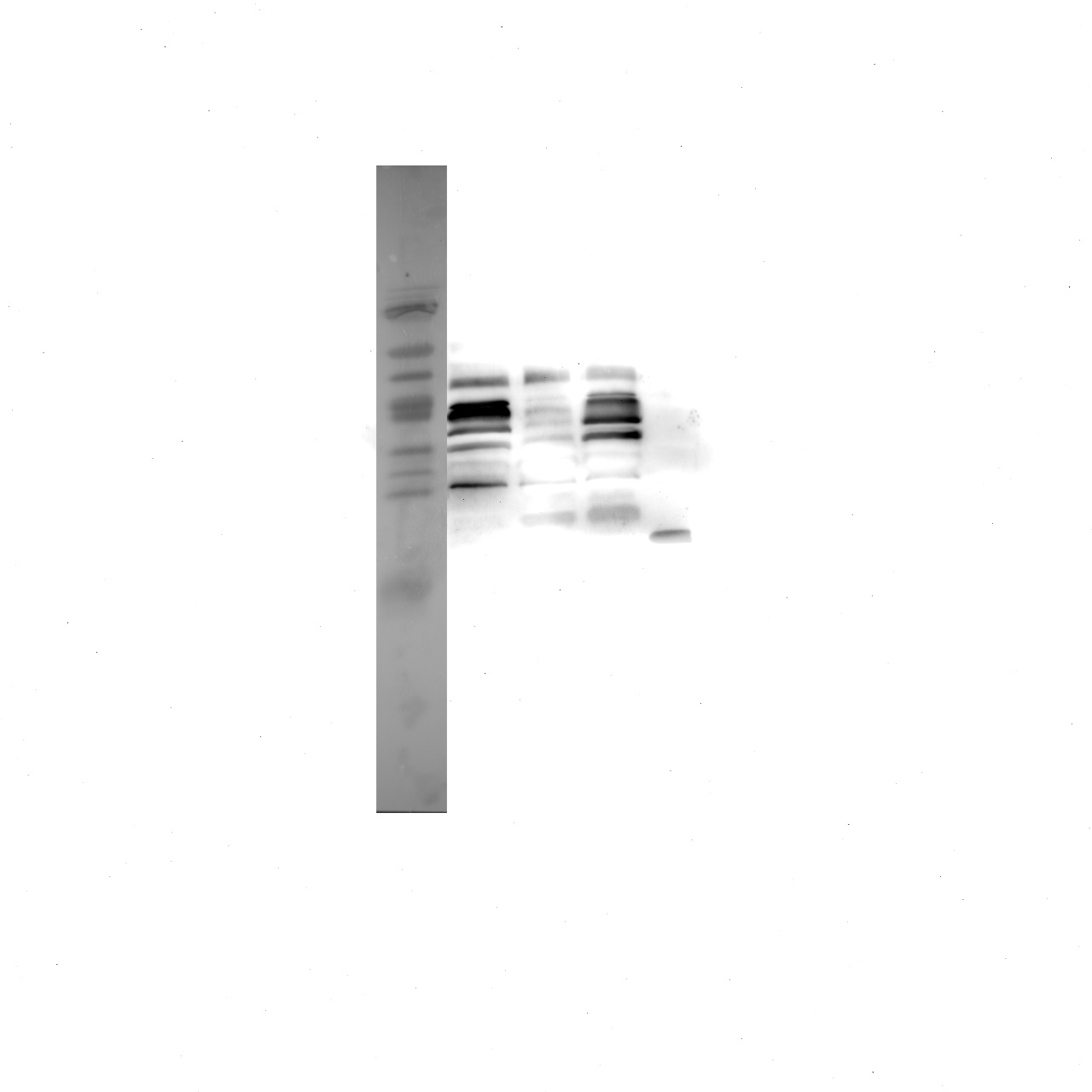


Figure 2b-2


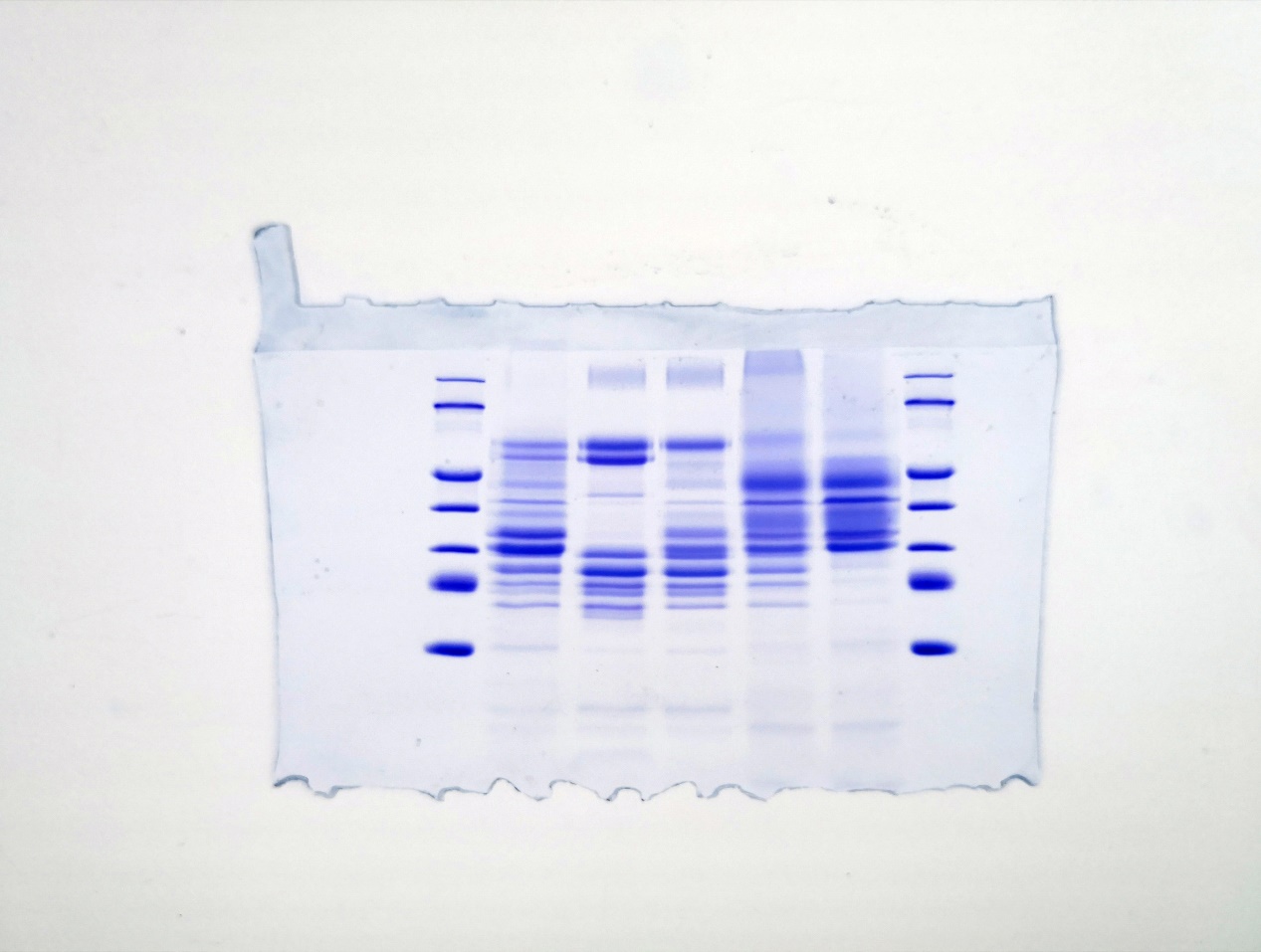


Figure 2c-1


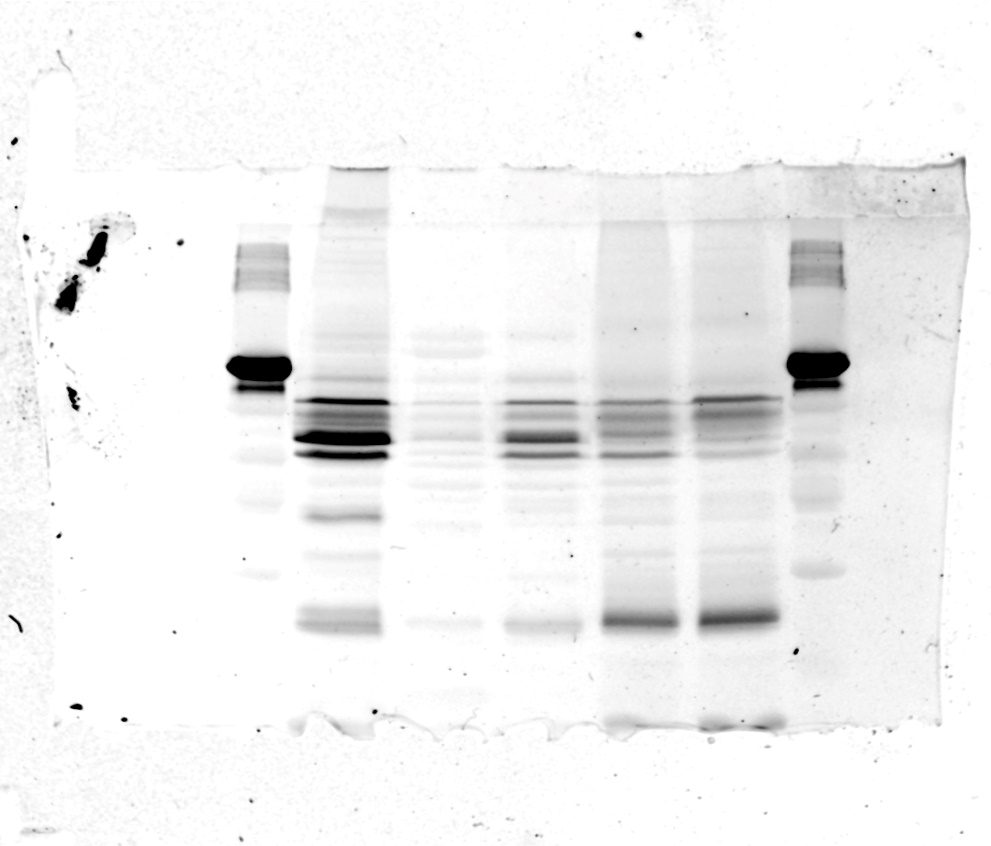


Figure 2c-2

Supplement: Supplementary file 4 — Source Data [file 41467_2021_21362_MOESM4_ESM.zip › Source Data.docx]
